# Differences in care between younger and older patients in the 2019 English national memory service audit

**DOI:** 10.1192/bjb.2021.104

**Published:** 2022-12

**Authors:** Laura D. Cook, Helen Souris, Jeremy D. Isaacs

**Affiliations:** 1 NHS England/NHS Improvement (London) Dementia Clinical Network, London, UK; 2 St George's University Hospitals NHS Foundation Trust, London, UK

**Keywords:** Dementia, psychosocial interventions, clinical governance, outpatient treatment, imaging

## Abstract

**Aims and method:**

This paper analyses how practice varied between patients aged <65 and ≥65 years in the 2019 UK national memory service audit.

**Results:**

Data on 3959 patients were analysed. Those aged <65 (7% of the sample) were less likely than those aged ≥65 to be diagnosed with dementia (23 *v.* 67%) and more likely to receive a functional, psychiatric or no diagnosis. Younger patients were more likely to have magnetic resonance imaging; use of dementia biomarkers was low in both groups. Frontotemporal dementia and functional cognitive disorder were diagnosed infrequently. Use of dementia navigators/advisors and carer psychoeducation was similar between groups; younger patients were less likely to be offered but more likely to accept cognitive stimulation therapy.

**Clinical implications:**

Memory services seeing younger people need expertise in functional cognitive disorder, alongside clinical skills and technologies to diagnose rarer forms of dementia. Further work is needed to understand why cognitive stimulation therapy is less frequently offered to younger people.

There are about 700 000 people living with dementia in England, a figure likely to increase owing to demographic ageing. The majority of patients being assessed for, and diagnosed with, dementia, are seen in community-based memory services, usually provided by National Health Service (NHS) mental health trusts. To better understand variation between providers, and as a tool for quality improvement, the NHS London Dementia Clinical Network developed a case-note based audit of memory services which was used in London in 2015, 2016 and 2019. For the 2019 round, services throughout England were invited to participate. Initial results comparing service-level data were published by NHS England and NHS Improvement.^1^ Here we present analysis of the pooled audit data-set. This provides a broad-based overview of memory service practice in England, with specific attention to how this varies by patient age.

## Method

An expert reference group consisting of primary and secondary care clinicians, memory service managers and commissioners convened by the London Dementia Clinical Network developed a ‘best practice’ clinical data-set for a pilot audit in 2015. This was refined for audit rounds one and two in 2016^2^ and 2019 respectively. The group reviewed existing standards and guidance, for example the Memory Service National Accreditation Programme (MSNAP) standards and National Institute for Health and Care Excellence (NICE) guidelines. The data-set covered the following areas: patient demographics, referral, assessment, investigation, diagnosis, treatment, follow-up and research participation.

For the 2019 audit, NHS England/NHS Improvement regional dementia clinical networks were invited to lead the process in their geographical areas. Regional networks then contacted memory services in their region to promote participation.

Participating memory services were asked to complete an anonymised case-note audit of 50 consecutive patients who underwent initial assessment from 1 January 2019. The audit tool recorded demographic information (age, gender and ethnicity) but not patient-identifiable data such as name, address, or hospital or NHS number. The guidance notes for the audit defined mild cognitive impairment (MCI) as ‘subjective experience of a decline from a previous level of cognitive functioning, accompanied by objective evidence of impairment that is not sufficiently severe to significantly interfere with independence in the person's performance of activities of daily living’. Service providers submitted anonymised data to their regional network between May and September 2019.

Regional networks transferred data to the London Dementia Clinical Network for analysis of the national data-set using Microsoft Excel. Statistical comparisons were performed using chi-squared tests.

## Results

Five NHS England/NHS Improvement regions participated in the audit: North East and Yorkshire; East of England; Midlands; London; and South East. Eighty-five memory services participated in the audit, contributing data on 3978 patients in total (mean age 79 years (range 30–102), 57% female). In 19 cases the patient's age was not provided.

### Diagnosis

Younger patients were much less likely to be diagnosed with dementia and more likely to receive a functional or psychiatric diagnosis or no diagnosis. Breakdown by broad diagnostic category is given in Table 1.

**Table 1:** Breakdown of main diagnostic categories by age cohort (<55, <65, ≥65 years)^a^

	All patients (*n* = 3707), *n* (%)	Patients aged <55 (*n* = 50), *n* (%)	Patients aged <65 (*n* = 243), *n* (%)	Patients aged ≥65 (*n* = 3464), *n* (%)	*P*
Dementia	2386 (64)	7 (14)	57 (23)	2329 (67)	<0.001
Mild cognitive impairment	634 (17)	6 (12)	41 (17)	593 (17)	0.921
Primary psychiatric	80 (2)	7 (14)	27 (11)	53 (2)	<0.001
Functional cognitive disorder	16 (0.4)	4 (8)	11 (5)	5 (0.1)	<0.001
Other diagnosis	205 (6)	12 (24)	32 (13)	173 (5)	<0.001
No diagnosis given	386 (10)	14 (28)	75 (31)	311 (9)	<0.001

a. Column totals exclude patients for whom the diagnosis field was left blank. *P*-values refer to comparison between between patients aged <65 and ≥65.

### Use of investigations

In the entire cohort, 439 patients (11%) underwent diagnostic neuropsychological assessment. Of these, where a diagnosis was given, 181 (46%) were diagnosed with dementia and 84 (21%) were diagnosed with MCI. In 86 cases (26%) it was reported that no diagnosis was given. Overall, younger patients were more likely to undergo diagnostic neuropsychological assessment than older patients (Table 2).

**Table 2:** Neuropsychology and neuroimaging in patients aged <65 compared with patients aged ≥65 years^a^

	Patients aged <65			Patients aged ≥65		
	Dementia	MCI	Other diagnosis	Dementia	MCI	Other diagnosis
Diagnostic neuropsychology performed, *n* (%)	15 (26)	11 (28)	48 (34)	166 (7)	73 (12)	79 (15)
*P*				<0.001	0.005	<0.001
Neuroimaging performed before or after assessment	46 (85)	36 (88)	104 (76)	1824 (84)	457 (80)	323 (65)
*P*				0.870	0.240	0.027
Where scan requested by memory service, MRI rather than CT, *n* (%)	20 (61)	13 (42)	44 (53)	330 (22)	106 (27)	67 (25)
*P*				<0.001	0.084	<0.001

MCI, mild cognitive impairment; MRI, magnetic resonance imaging; CT, computed tomography.

a. *P*-values refer to comparison between patients aged <65 and ≥65.

Among those diagnosed with dementia, more than three times as many with young-onset dementia (YOD) (defined here as age <65 years at initial assessment) received a diagnostic neuropsychology assessment compared with those with late-onset dementia (LOD) (age ≥65 at initial assessment) (Table 2). In both younger and older cohorts, patients with non-dementia diagnoses were most likely to undergo neuropsychological evaluation, followed by those diagnosed with MCI. Patients diagnosed with dementia were least likely to undergo neuropsychological evaluation (Table 2).

**Fig. 1:**
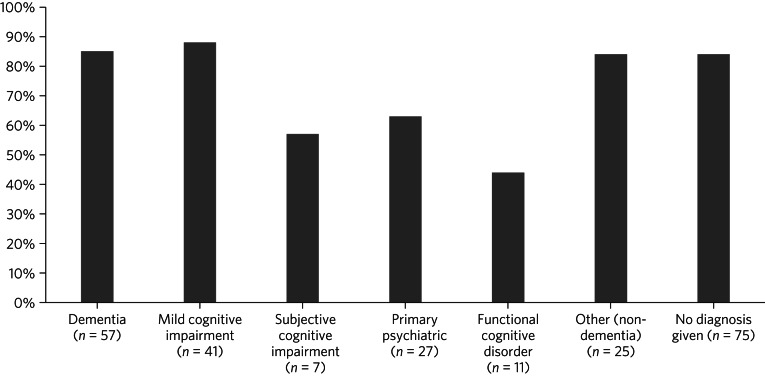
Percentage of patients aged <65 years undergoing brain imaging, by diagnostic group.

The proportion of younger and older patients undergoing brain imaging as part of or prior to memory service assessment was similar (Table 2). However, younger patients were more likely to be referred for a magnetic resonance imaging (MRI) scan (as opposed to a computed tomography (CT) scan), particularly among those diagnosed with dementia. A majority of patients receiving non-dementia or MCI diagnoses underwent brain imaging, including 76% of those aged <65 (Table 2). Scanning rates by diagnosis among patients aged <65 are shown in Fig. 1. Patients with functional cognitive disorder and primary psychiatric diagnoses had the lowest scanning rates.

### Specialist investigations

Three patients who received a diagnosis of YOD (6%) had a specialist investigation; two had positron emission tomography (PET) scans and one a dopamine transporter (DAT) scan. One younger patient diagnosed with MCI (3%) had a specialist investigation (a PET scan). Four younger patients who were not diagnosed with dementia or MCI (3%) had a specialist investigation: 2 had DAT scans and 2 had PET scans.

Of the patients diagnosed with LOD, 51 (2%) had a specialist investigation: 36 had DAT scans, 10 had PET scans, 3 had single-photon emission computerised tomography (SPECT) scans, 1 had cerebrospinal fluid (CSF) examination and 1 had a PET scan and CSF examination. Five people aged ≥65 diagnosed with MCI (1%) had specialist investigations: three had a PET scan, one had a SPECT scan and one had a DAT scan. Nine older people who were not diagnosed with dementia or MCI (2%) had a specialist investigation: five had a PET scan and four had a DAT scan.

### Demography of young-onset versus late-onset dementia

Compared with patients with late-onset dementia (LOD), those with young-onset dementia (YOD) were more likely to be male, to smoke and to exceed recommended alcohol consumption and less likely to live alone (Table 3). There were no differences between the two groups in the location of the assessment (patient's home versus clinic) or in the proportion of patients asked about hearing, vision or falls (data not shown).

**Table 3:** Demographics, subtype diagnosis and care, comparing people with young-onset (aged <65) and late-onset dementia (aged ≥65)^a^

	Young-onset dementia group, *n* (%)	Late-onset dementia group, *n* (%)	*P*
Female	23 (41)	1372 (59)	0.007
BAME	8 (15)	266 (12)	0.206
Live alone	12 (22)	842 (38)	0.021
Consume >14 units of alcohol per week	8 (19)	96 (6)	<0.001
Smoke	11 (23)	155 (8)	<0.001
Dementia subtype			
Alzheimer's disease	21 (37)	1067 (46)	0.179
Vascular dementia	11 (19)	392 (17)	0.620
Alcohol-related dementia	7 (12)	10 (0.4)	<0.001
Unspecified dementia	5 (9)	138 (6)	0.370
Mixed dementia	4 (7)	594 (26)	0.001
Other dementia	4 (7)	15 (1)	<0.001
Frontotemporal dementia	4 (7)	7 (0.3)	<0.001
Parkinson's disease dementia	1 (2)	62 (3)	0.668
Dementia with Lewy bodies	0 (0)	44 (2)	0.293
Cognitive stimulation therapy (CST)			
CST offered	12 (21)	754 (34)	0.042
Of those offered, number declining	2 (17)	342 (45)	0.047
CST deemed not appropriate	31 (54)	877 (39)	0.023
No service available	14 (25)	590 (27)	0.735
Care navigation/dementia advisor			
Received care navigation	44 (77)	1829 (80)	0.643
Carer psychoeducation			
Carer psychoeducation offered	16 (29)	865 (39)	0.729
Of those offered, number declining	2 (13)	259 (30)	0.121
Carer education deemed not appropriate	20 (36)	694 (32)	0.451
No service available	19 (35)	639 (29)	0.378
Research participation			
Research participation offered	20 (35)	834 (37)	0.764
Of those offered, number declining	5 (25)	442 (53)	0.013

BAME, Black, Asian and minority ethnic.

a. *P*-values refer to comparison between patients aged <65 and ≥65.

### Subtype diagnosis

A higher proportion of people with YOD were diagnosed with alcohol-related dementia, frontotemporal dementia (FTD) and ‘other dementia’ compared with those with LOD (Table 3). A lower proportion were diagnosed with mixed dementia. No patients aged under 65 were diagnosed with dementia with Lewy bodies.

### Waiting times and treatment

The mean wait from referral to diagnosis was 12 weeks for patients with YOD and 13 weeks for those with LOD.

There were no significant differences between YOD and LOD in terms of numbers of patients with eligible subtypes (i.e. a diagnosis of Alzheimer's disease, mixed dementia, dementia with Lewy bodies or Parkinson's disease dementia) prescribed cholinesterase inhibitors and/or memantine (data not shown).

Cognitive stimulation therapy (CST) was deemed not appropriate in a significantly higher proportion of the YOD group, leading to a significantly lower proportion being offered CST compared with the LOD group (Table 3). Where CST was offered, older people were significantly more likely to decline it. Similar proportions of individuals in both groups were offered care navigation, carer psychoeducation and consent to be contacted for research. The YOD group were more likely to accept research participation than the LOD group.

## Discussion

The development of memory services in England as a bespoke pathway for assessment of people with suspected dementia, separate from community mental health and neurology services, was promoted by the 2009 National Dementia Strategy.^3^ The subsequent expansion of memory services was supported by an optional quality improvement infrastructure (Memory Services National Accreditation Programme) led by the Royal College of Psychiatrists. This was not accompanied by a national audit data-set, in contrast to the National Audit of Dementia, which examines care in acute hospitals. Notably, national surveys of memory services conducted in 2013 and 2014 did not include patient-level data. This first large-scale audit of memory services in England provides important insights into diagnostic and immediate post-diagnostic practices, including how these differ by age.

Our data show that only a small proportion of younger people referred to memory services have dementia (14% of those aged <55, 23% of those aged <65), in accordance with our previous audit round in London and findings from Cambridgeshire.^2,4^ English memory services diagnose 1 person with YOD for every 40 with LOD. This makes YOD a small fraction of memory service workload and might limit the ability of memory service clinicians to build expertise in this condition, which the Royal College of Psychiatrists has identified as a priority.^5^ Whether younger people are better served by specialist YOD services or can be appropriately supported within ageless memory services remains a subject of debate.^4,6^ Either way, the higher prevalence of psychiatric disorders in younger people referred to memory services suggests that psychiatry-led services are an appropriate initial point of referral provided they include cognitive neurologists or have good links to specialist neurological services.

Do the audit data suggest that memory services are underskilled in YOD and rarer dementias? Population studies suggest that FTD accounts for 10% of YOD and just under 3% of LOD.^7^ Our finding that 7% of patients with YOD and 0.3% of patients with LOD were diagnosed with FTD likely reflects either referral bias or underdiagnosis.

Younger individuals presenting to primary care with atypical symptoms such as language decline or personality change might be referred to neurology services or community mental health services and therefore not appear in memory service cohorts. Nevertheless, pathological series suggest that over half of FTD cases might be clinically misdiagnosed, most commonly as Alzheimer's disease.^8^

In the absence of disease-modifying therapy, the impact of subtype misdiagnosis on disease progression is relatively limited; nevertheless, dementia subtypes differ in terms of aetiology, prognosis and response to symptomatic treatment. Counselling advice given to patients and families is therefore potentially misleading if based on an inaccurate subtype.

Furthermore, nearly one in ten individuals with YOD in this sample were diagnosed with ‘unspecified dementia’, compared with 6% of those with LOD. Dementia in younger people is more likely to be caused by a single neuropathological process and confounding factors such as frailty are less prevalent. A specific subtype diagnosis should be attempted wherever possible, particularly given the higher rate of genetic mutations in YOD, so that appropriate counselling can be given to patients and families.

The audit demonstrates the resource implications of assessing individuals with YOD, with higher proportions of these patients being referred for diagnostic neuropsychology and MRI scans. In the audit organisational questionnaire, 87% of services reported that they could refer patients for DAT scans, 77% for PET scans and 56% for CSF examination. However, the low rate of functional and biomarker-based investigations in both younger and older patients suggests that these referral pathways are rarely used. Routine use of biomarkers in dementia diagnosis remains contested.^9^ Nevertheless, our data suggest a system largely unfamiliar with molecular diagnosis in dementia and in need of significant additional educational and financial resources should this become necessary.^10^

That 31% of patients aged <65 and one in four of all patients who underwent neuropsychological assessment did not receive any diagnosis suggests that opportunities for therapeutic intervention may be being missed. Although not everyone referred to secondary care has a cognitive or psychiatric disorder, that people choose to attend memory service assessment suggests that they have at least some unmet need. A recent systematic review reported that people seeking help for subjective cognitive symptoms have high rates of psychological morbidity and should not be dismissed as ‘worried well’.^11^ It is possible that patients coded in the audit as ‘no diagnosis given’ received a diagnosis after the audit census date. However, given that services completed the audit 4–8 months after patients were first seen, this would suggest that a significant minority of memory service patients, particularly those aged <65, are waiting many months for a diagnosis.

Although it is important not to pathologise people who present with mild symptoms not meeting thresholds for psychiatric diagnosis, giving meaning to people's symptoms usually requires healthcare professionals to give those symptoms a label or to provide an explanatory formulation. That this is often not possible even following a detailed neuropsychological assessment suggests that the framing of assessment in memory services as positioning patients on a deterministic pathway from ‘normal’ to MCI to dementia risks missing non-dementia contributors to cognitive symptoms.

Functional cognitive disorder (FCD) is an increasingly recognised cause of distressing cognitive symptoms.^12^ The use of this diagnosis for only 5% of patients aged <65 and 0.1% of those aged ≥65 in our sample suggests underrecognition or coding issues. FCD does not feature in the UK edition of SNOMED CT, the coding catalogue used by NHS Digital; the closest match is ‘Dissociative neurological symptom disorder co-occurrent with cognitive symptoms’, which memory service clinicians are unlikely to use, as ‘dissociation’ is rarely the lead symptom in FCD.

The key features of FCD can only be elicited though careful clinical observation;^13^ it cannot be ruled in by investigations such as brain imaging or cognitive instruments. Interestingly, use of FCD as a diagnostic label was associated with a lower scanning rate, suggesting that its increased use might help reduce over-investigation.

Similarly, the proportion of younger people in this sample given a diagnosis of MCI seems too high, given that MCI was developed to define people considered at high risk of progressing to dementia, rather than as a generic term for cognitive symptoms not meeting threshold for dementia diagnosis.^14^ Where used inappropriately, MCI risks patients being given an inappropriately adverse prognosis and missing out on personalised psychotherapeutic interventions for FCD and psychiatric causes of cognitive symptoms. Conversely, lack of confidence in diagnosing dementia in younger people might lead to inappropriate use of MCI as a temporising measure, which has the effect of delaying diagnosis and intervention. This would be particularly injurious to younger people with dementia, who generally endure longer times from symptom onset to diagnosis than older people.^15^ In our view, MCI is most appropriately used not in isolation but as part of a formulation, in which likely contributory factors are cited.

In this audit, waiting times were similar for both younger and older people diagnosed with dementia. However, the audit only looked at waiting times following referral from primary care rather than from symptom onset. Longer duration from onset to diagnosis in YOD could still be caused by delayed symptom recognition and referral into secondary care.

Although our sample of people with YOD was relatively small, some potentially useful epidemiological observations emerge. People with YOD were nearly three times more likely to be smokers than those with LOD. Smoking prevalence among those with YOD was higher than in the general population.^16^ Smoking is a known risk factor for dementia,^17^ and vascular disease in general increases the risk of YOD.^18^

Alcohol consumption among individuals with LOD in our sample was broadly in line with population data, but was higher among those with YOD than in the general population,^19^ reflecting the known association between heavy alcohol use and dementia.^15^

In a quarter of cases, CST was not available for people with YOD, and in services where it was offered it was felt not to be appropriate for 54% of patients. However, when CST was offered to those with YOD, only 17% declined, compared with 45% in the LOD group. More work is required to understand why CST is either unavailable or felt to be inappropriate in such a large proportion of the YOD population. It is possible that memory service clinicians assumed that the content of the CST sessions would not be age appropriate for the younger population. Some services offer cognitive rehabilitation or occupational therapy to support functional ability in working-age patients. YOD-specific peer support groups are also available in some areas. These activities may be preferred to standard CST offered by memory services, which is often aimed at older people. However, clinicians should not rule out the possibility that a younger patient might accept CST.

Carer psychoeducation was not available for just over a third of individuals with YOD. Where offered, only 13% declined it, compared with 30% in the LOD group, although the small number in the YOD group meant that this difference did not reach statistical significance. There may be an assumption that the carer of a person with YOD might have time constraints related to being in employment or other caring responsibilities that preclude engagement. However, there may be an increased need for support among younger carers precisely because they typically have existing commitments and because of the extreme emotional, practical and financial dislocation associated with YOD.^3^

There were no discernible differences between YOD and LOD with respect to acceptance of care navigation. However, clinicians report difficulties finding age-appropriate resources and people with YOD and their carers report that services are unable to meet their personal and psychological needs.^20^

That people with LOD were less likely to accept evidence-based post-diagnostic interventions suggests that these are not always offered in a user-friendly way to the older population. Barriers to older people engaging in CST and carer psychoeducation include limited accessibility, travel costs, frailty or lack of a carer to accompany the patient. Commissioners and providers must ensure that interventions for which there is clear evidence of clinical and cost-effectiveness, and which are endorsed in national guidelines, are made available in ways that suit the needs of the population in whom they are indicated.

The audit results indicate that people with YOD are more inclined to engage with research participation than those with LOD. This likely reflects their greater mobility, as research centres are often regionally based. However, it also reflects the general underrepresentation of older patients in biomedical dementia research. This is problematic given that people aged 80 and over form the majority and the fastest growing cohort of those with dementia.

### Implications

This national memory service audit provides an unprecedented snapshot of dementia diagnosis and care in England. It reveals a sector in which clinical pragmatism is dominant, with minimal uptake of biomarker-based investigations. Despite a strong emphasis on clinically led assessment, FTD is probably being underdiagnosed and too many younger people are not receiving a subtype diagnosis, while patients with functional and psychiatric diagnoses are probably being mis-labelled.

Memory services operate within a constrained resource environment. Yet patients with functional and psychiatric diagnoses are probably receiving more investigations than required. Cost-effective interventions such as CST and carers’ psychoeducation remain patchily available.

The audit methodology presented here has been adopted by the National Audit of Dementia, which has been commissioned by the Healthcare Quality Improvement Partnership (HQIP) to conduct a further national memory service audit round in 2021. The COVID-19 pandemic has had an impact on quality improvement programmes aimed at addressing unwarranted variation revealed by the 2019 audit. Nevertheless, greater awareness of our findings should hopefully translate into improvements in future audit rounds, alongside the inevitable changes in practice driven by the pandemic.^21^

## Data Availability

The complete audit data are not publicly available owing to restrictions to prevent identification of individual services. The pooled data that support the findings of this study are available from the corresponding author on reasonable request.

## About the authors

**Laura D. Cook**, Clinical Programme Lead, NHS England/Improvement (London) Dementia Clinical Network, London, UK. **Helen Souris**, Clinical Project Manager, NHS England/Improvement (London) Dementia Clinical Network, London, UK. **Jeremy D. Isaacs**, Clinical Director, NHS England/Improvement (London) Dementia Clinical Network, and Consultant Neurologist, Department of Neurology, St George's University Hospitals NHS Foundation Trust, London, UK.
